# Predictive factors for medical resource utilization among Taiwan’s psychiatric inpatients

**DOI:** 10.1186/1472-6963-14-S2-P68

**Published:** 2014-07-07

**Authors:** Chin-Ming Lin, Chung-Yi Li

**Affiliations:** 1 Department of Healthcare Information and Management, Ming Chuan University, Taoyuan County, Taiwan; 2 Department and Graduate Institute of Public Health, College of Medicine, National Cheng Kung University, Tainan City, Taiwan

## Background

Taiwan launched a single-payer National Health Insurance program. The prevalence of psychiatric disorders has increased gradually since the implementation of this program. This could be because the national health insurance removed barriers to health care for the newly insured, enabling them more access to that health care. Determining the factors that contribute to medical utilization for psychiatric patients could provide evidence for how the mental health system should target resources and care management to avoid social and cost crises. The purpose of the study is to explore the predictors for medical utilization for psychiatric inpatients.

## Materials and methods

The present study consisted of 974 inpatients discharged from a public psychiatric hospital in Taiwan in 2005. Demographic characteristics, discharge diagnoses, and medical utilizations were retrieved from the inpatient claim data of the National Health Insurance Database. Multivariate logistic regression models were performed to identify significant predictors for length of stay (LOS) and medical charge.

## Results

A median LOS of 40.0 days and median medical charges of US $3104.10 were reported. A greater likelihood of high medical utilization was found among patients who were exempt from making co-payments, were diagnosed with schizophrenia or depression, had a co-morbidity factor, or who came from emergency visits. There was no significant association between re-admission and increased LOS or medical charges.

## Conclusions

The study found that demographics, disease characteristics and insurance policies were all associated with high medical utilization. The study may be useful in future assessment of whether the medical resources available in treating psychiatric patients are optimally allocated.

